# Effects of vaginal administration of conjugated estrogens tablet on sexual function in postmenopausal women with sexual dysfunction: a double-blind, randomized, placebo-controlled trial

**DOI:** 10.1186/s12905-020-01031-4

**Published:** 2020-08-12

**Authors:** Thanapob Bumphenkiatikul, Krasean Panyakhamlerd, Thanittha Chatsuwan, Chai Ariyasriwatana, Ammarin Suwan, Charoen Taweepolcharoen, Nimit Taechakraichana

**Affiliations:** 1 Division of Reproductive Medicine, Department of Obstetrics and Gynecology, Faculty of Medicine, Chulalongkorn University, 1873 Rama IV Road, Bangkok, 10330 Thailand; 2 Department of Microbiology, Faculty of Medicine, Chulalongkorn University, Bangkok, 10330 Thailand; 3 Division of Gynecologic Cyto-Pathology, Department of Obstetrics and Gynecology, Faculty of Medicine, Chulalongkorn University, Bangkok, 10330 Thailand; 4 Department of Medical Education and Clinical Research Center, Bumrungrad International Hospital, Bangkok, 10110 Thailand

**Keywords:** Sexual dysfunction, Vulvovaginal atrophy, Genitourinary syndrome of menopause, Dyspareunia, Hormonal therapy, Vaginal pH, Vaginal maturation index, The female sexual function index

## Abstract

**Background:**

Female sexual dysfunction (FSD) is prevalent in women with genitourinary syndrome of menopause (GSM). Vaginal estrogen is effective GSM treatment. This study was primarily aimed to evaluate the effects of vaginal administration of conjugated estrogens tablet on postmenopausal FSD using the Female Sexual Function Index (FSFI). Secondary aims were to evaluate vaginal pH, Vaginal Maturation Value (VMV), Normal Flora Index (NFI) and Most Bothersome Symptoms (MBS) changes.

**Methods:**

A double-blind trial was conducted in postmenopausal women with FSD (FSFI ≤26.55). Sixty-seven participants were randomized into two arms; vaginally administered conjugated estrogens tablet (0.625 mg, daily for 3 weeks then twice weekly for 9 weeks, *n* = 33), or placebo (*n* = 34).

**Results:**

There was no significant improvement of FSFI observed in estrogens arm compared to placebo in each domain and overall index (*p* = 0.182). The estrogens significantly improved vaginal pH and VMV, toward more acidity (*p* = < 0.001), higher VMV (*p* = < 0.001) and more superficial cells (*p* = < 0.001). We observed no significant difference in NFI and MBS between arms (*p* = 0.282, 0.182).

**Conclusion:**

We found no significant changes in FSFI, NFI, and MBS, but significant improvement in vaginal pH and VMV in postmenopausal women with FSD treated with vaginally administered conjugated estrogens tablet. Few side-effects were reported.

**Trial registration:**

Thai Clinical Trial Registry identification number TCTR20180219001, prospectively registered since 2018-02-19 11:33:21.

## Background

Female sexual dysfunction (FSD) is an important condition which prevalence is difficult to estimate [1] and thought to be underreported. A 2005-report showed that 38 to 63% of women all over the world suffered from FSD [2]. The prevalence of FSD increases with age [3]. In a southern province of Thailand, the proportion of postmenopausal women diagnosed with sexual dysfunction based on FSFI overall scores of 26.5 or less was 82.2% [4].

According to DSM-5 criteria, FSD is classified into three major categories: female sexual interest/arousal disorders (FSIAD), female orgasmic disorder and genitopelvic pain/penetration disorder (GPPPD) [5]. It was postulated that there were links between all three categories in etiology and also treatment, i.e., if the patient is treated for GPPPD, there might be an improvement in FSIAD and vice versa [6].

GPPPD was formerly subdivided into dyspareunia and vaginismus in DSM-IV criteria, but later the two subdivisions were merged together [5]. Dyspareunia in postmenopausal period is generally due to genitourinary syndrome of menopause (GSM), a novel broader term for vulvovaginal atrophy (VVA), encompassing three domains of menopausal change, i.e., sexual, urinary and genital symptoms [7].

GSM is a condition resulting from the hypoestrogenic vaginal epithelium. With less glycogen storage in vaginal epithelium, the vaginal mucosa becomes thinner and vasculature become minimal, causing less transudate in the vagina*. Lactobacillus spp.*, normal flora of vaginal microbiome becomes lack of substrate to produce lactic acid to maintain vaginal acidity; hence, the menopause vagina becomes dry, pale, thin, basic, and colonized with pathologic bacteria instead of *Lactobacillus spp.* as compared to premenopausal women [8]. This phenomenon is the cause of dyspareunia during postmenopause. The gold standard of GSM treatment is hormonal therapy, preferably with local estrogen application to avoid unwanted systemic effect particularly when there is no FDA approved indication for systemic hormone therapy [9–12].

There are several preparations of vaginal estrogen for treating GSM. Conjugated estrogens tablet (0.625 mg) has been listed in the Thai National List of Essential Medicines 2018 for use as female sex hormone while conjugated estrogens vaginal cream listed for Treatment of vaginal and vulval conditions [13]. However, conjugated estrogens vaginal cream is not available in Thailand, driving patients and physicians to seek alternative treatment for GSM.

In the past decades, there have been questions of whether the official labelled oral estrogens can be used to treat GSM via vaginal administration. Several studies were conducted both in postmenopausal and premenopausal women showing that the benefits from vaginal administration of several oral estrogens were not inferior to standard oral administration [14–16]. To our knowledge, there were no published studies on the use of the country-wide available conjugated estrogens tablet in improving FSD with vaginal administration. Therefore, the primary objective of the present study was to evaluate the effects of vaginal administration of conjugated estrogens tablet on sexual function in postmenopausal women with FSD using the Female Sexual Function Index (FSFI). The secondary objectives were to evaluate changes in vaginal pH, Vaginal Maturation Value (VMV), Normal Flora Index (NFI) and any possible side effects of the treatment. If vaginal administration of conjugated estrogens tablet could improve sexual function in postmenopausal women with FSD, this low-cost, easy-to-use and widely available estrogens would be an interesting treatment option to improve this prevalent condition.

## Methods

### Design

The study was designed as a single-center, prospective, double-blind, randomized, placebo-controlled trial, which randomized participants into two groups in a 1:1 ratio. The study adhered to CONSORT guidelines and was approved by the Institutional Review Board of the Faculty of Medicine, Chulalongkorn University (IRB No. 039/2561). The study was also reviewed by the Thai Clinical Trial Registry Committee and prospectively approved for registration since 2018-02-19 11:33:21 and Thai Clinical Trial Registry identification number TCTR20180219001. After approval, participants were included from August 2018 to March 2019. A thorough explanation of the study details was given to all enrolled women. Informed written consent was obtained prior to the start of the study.

### Patient recruitment

Literate Thai women aged 45–70 years who experienced spontaneous menopause attending the General Gynecologic Clinic, Climacteric and Gender Health Clinic at King Chulalongkorn Memorial Hospital were recruited. Menopause was defined according to WHO criteria as the cessation of the period for at least 12 consecutive months [17].

### Inclusion and exclusion criteria

These women were asked for their vaginal atrophy symptoms and other detailed histories. Participants were included if they reported at least one self-assessed vaginal atrophy symptom in moderate or severe intensity and reported engaging in penile-vaginal penetrative sexual intercourse at least once a month. Those with pathological or surgical causes of menopause, using menopausal hormone therapy (MHT) or non-hormonal treatment that might affect the vaginal epithelium, having abnormal vaginal bleeding/discharge without prior appropriate investigation and treatment, cervical or vaginal surgical history within the previous 3 months, history of sex steroid hormone use 2 months prior to study, history of psychiatric disorders or having partner with sexual dysfunction, contraindications for MHT including personal or family history of estrogen-related cancer, severe liver or kidney diseases and any suspected allergy to MHT, were excluded from the study. The recruited subjects were then asked to complete a 19-item Thai version of the Female Sexual Function Index questionnaire, and those that scored > 26.55 points were then excluded as having no FSD. The Thai version of the Female Sexual Function Index questionnaire was validated in previous study among Thai postmenopausal women, aged 40–60 years, with high reliability coefficients and internal consistency (r = 0.79–0.86, Cronbach’s alpha value = 0.82) [18]. After pelvic examination and test for vaginal pH, those with pH more than 5 were qualified for the study as they were likely to have poor estrogenic vaginal mucosa and might benefit from the treatment.

### Randomization and blinding

A computer generated block of four randomizations was used to randomize participants into two groups in a 1:1 ratio. The allocation and concealment of placebo and the drugs into identical opaque envelopes were done by a nurse who was not involved in contacting with patients or analyzing any data, thus blinding investigators, assessors, cytologist, microbiologist and all participants.

### Sample size justification

The sample size of this study was estimated based on our pilot study, conducted with 10 participants enrolled in both arms. The following formula was used for comparing continuous data in a randomized controlled trial [19].

$$ {\displaystyle \begin{array}{c}{n}_{trt}=\frac{{\left({z}_{1-\frac{\alpha }{2}}+{z}_{1-\beta}\right)}^2\;\left[\sigma {}_{trt}{}^2 + \frac{\sigma {}_{con}{}^2}{r}\right]}{\varDelta^2}\\ {}r=\frac{n_{con}}{n_{trt}},\varDelta ={\mu}_{trt}-{\mu}_{con}\end{array}} $$

According to our pilot study, μtrt (mean value) and σtrt (standard deviation) of total FSFI at 12 week follow up in treatment group were 26.8 and 5.7, respectively; μcon and σcon of total FSFI at 12 week follow up in control group were 23.0 and 4.7, respectively. The ratio between groups (r) was set as 1 for 1:1. With using α = 0.05 and β = 0.2, the sample size needed for this study was 30 participants per group. Predicting a 10% drop out rate, the sample size was increased to 33 participants per group.

### Intervention

After completion of history taking and filling all the questionnaires, participants who met the inclusion criteria mentioned above were asked to undergo a pelvic examination, all conducted by single investigator (T.B.). A dry speculum was inserted without lubrication. A pH-indicator strip (pH range 0–14, Merck, Germany) was placed over the upper to the middle third of the lateral vaginal wall with contact time between the pH-indicator strip and the examined vaginal wall for 3 s.

Two dry cotton buds were used to scrape contralateral vaginal wall. Each cotton bud was smeared onto each different glass slide. One slide was left to air dry and sent for evaluation of Normal Flora Index (NFI) with Gram staining by single microbiologist (T.C.), unaware of treatment allocation, participant symptoms and characteristics. NFI, representing vaginal microenvironment, consisted of 4 parameters, i.e., the number of lactobacilli, pathogenic microorganisms, leukocytes, and vaginal pH. Each parameter was graded on a 4-point scale (Table 1). Pathogenic microorganisms included Gardnerella, Bacteroides, Mobiluncus and gram-variable bacilli [21, 22].

**Table 1:** The Female Sexual Function Index scoring system [20]

*Domain*	*Questions*	*Score range*	*Factor*	*Minimum score*	*Maximum score*
desire	1, 2	1–5	0.6	1.2	6
arousal	3, 4, 5, 6	0–5	0.3	0	6
lubrication	7, 8, 9, 10	0–5	0.3	0	6
orgasm	11, 12, 13	0–5	0.4	0	6
satisfaction	14, 15, 16	0 (or 1) -5	0.4	0.8	6
pain	17, 18, 19	0–5	0.4	0	6
Full-scale score range				2	36

The other slide was fixed with 95% ethanol solution for 30 min and sent for staining in accordance with Papanicolaou test. The slide was evaluated for Vaginal Maturation Value (VMV) by single cytologist (C.A.), unaware of treatment allocation, participant symptoms/characteristics. VMV, calculated from the formula: (% of intermediate cells × 0.5) + (% of superficial cells × 1), is considered as a surrogate of vaginal epithelium estrogen status [23, 24–26].

The number of lactobacilli, pathogenic microorganisms and VMV was evaluated under the microscope with 1000x magnification (HPF). The number of leukocytes was evaluated under the microscope with 400x magnification.

Participants were then examined with transvaginal ultrasonography for baseline endometrial thickness [Samsung SONOACE R7, 2D imaging mode, grayscale 256 (8 bits), EVN4–9 probe 3.5 MHz, single operator (T.B.)]. Blood samples were taken for baseline hematocrit, SGOT, SGPT, alkaline phosphatase, total cholesterol, LDL, HDL, triglycerides and estradiol level. The samples were analyzed immediately, or stored at 4 degrees Celsius until analysis no more than 24 h later. All blood sample analyses were done with routine laboratory testing platform for research projects at the King Chulalongkorn Memorial Hospital, strictly adhering to the manufacturers’ protocol and in accordance with the laboratory’s standard operating procedures for good laboratory practice.

After the recruitment process, participants were randomized into the conjugated estrogens or the placebo groups. The estrogens or placebo was given in numbered as identical opaque envelopes with instruction leaflet. The treatment arm was conjugated estrogens tablet (EstromonTM, 0.625 mg). The placebo arm was lactose 90%, polyvinyl pyrrolidone K30 5%, magnesium stearate 3%, talcum 1% erythrosinelahe dye 1% and water which was evaporated completely during manufacturing. The placebo was made by the Faculty of Pharmaceutical Science, Chulalongkorn University and was visually identical to the conjugated estrogens tablet. No participants reported allergic reactions to lactose, talcum or any substances used in the study. Participants were required to insert each pill vaginally as deeply as possible every day for 3 weeks. After 3 weeks of participation, the investigator called to each participant to check on adherence, problems of drug administration or adverse reactions and willingness to continue the study, after which participants were advised to continue the study by inserting a pill vaginally twice weekly on every Monday and Friday night for the next 9 weeks. The regimen was extrapolated from recommendation for treating dyspareunia with conjugated estrogen vaginal cream [11].

After the 12th week of study, each participant came back for reevaluation of symptoms, filling out questionnaires, undergoing pelvic examination for vaginal pH, smear for NFI and VMV, transvaginal ultrasonography and blood samples.

### Outcome measures

The primary outcome of this study was the changes of FSFI of the two treatment arms. FSFI was one of the validated standard questionnaires frequently used for assessing female sexual function and quality of life in clinical trials or epidemiological studies concerning sexual study [20, 27]. Since there were no validated measurement tools specifically designed for female sexual function and dysfunction in menopausal population, and FSFI was frequently used in researches concerning female sexual function and quality of life in menopausal population, the index was selected for this study. The questionnaires consisted of 19 self-reporting rating-scale items, assessing 6 domains of sexual function; desire, arousal, lubrication, orgasm, satisfaction and pain. Each item has a scaled response ranging from 0 to 5 or 1 to 5; with higher scores representing better sexual function (Table 1). Each domain score is calculated by summation of scores from every item in the domain multiplied by the domain factor (i.e., 0.6 for desire, 0.3 for arousal and lubrication, 0.4 for orgasm, satisfaction and pain), thus the full score of each domain is 6. The possible full scores of total FSFI ranges from 2.0 to 36.0, with cut-off point of 26.55 or less considered FSD in premenopausal and postmenopausal women population [28]. However, there is no specific cut-off score for FSD in a population with GSM [29].

Secondary outcomes were changes in vaginal pH, VMV, NFI, and the Most Bothersome Symptoms (MBS). The MBS, also known as the vaginal atrophy symptoms consists of 4 symptoms; vaginal dryness, vaginal/vulvar irritation/itching, vaginal/vulvar soreness, and dyspareunia. Each symptom is self-graded by participants on a 4-point scale (0 = no symptom, 1 = mild, 2 = moderate, 3 = severe) [30]. Only those reported at least one self-assessed vaginal atrophy symptom in moderate or severe intensity were included. The safety parameters assessed were changes in hematocrit, SGOT, SGPT, alkaline phosphatase, total cholesterol, LDL, HDL, triglycerides, estradiol level and endometrial thickness.

### Statistical analysis

IBM SPSS™ statistics version 18.0 for Windows was used for statistical analysis. The treatment effect was evaluated with intention-to-treat analysis, with missing data assumed by multiple imputation method. Baseline demographic characteristics were presented using descriptive statistics; mean and standard deviation (SD), median and interquartile range (IQR), or number and percentage as appropriate. Primary and secondary outcome comparisons between groups were evaluated with Mann-Whitney U test or analysis of covariance (ANCOVA), with treatment arm as a fixed effect in the model and the baseline value used as a covariate, according to data distribution characteristics. *P-value* of less than 0.05 is determined statistically significant.

## Results

**Fig. 1:**
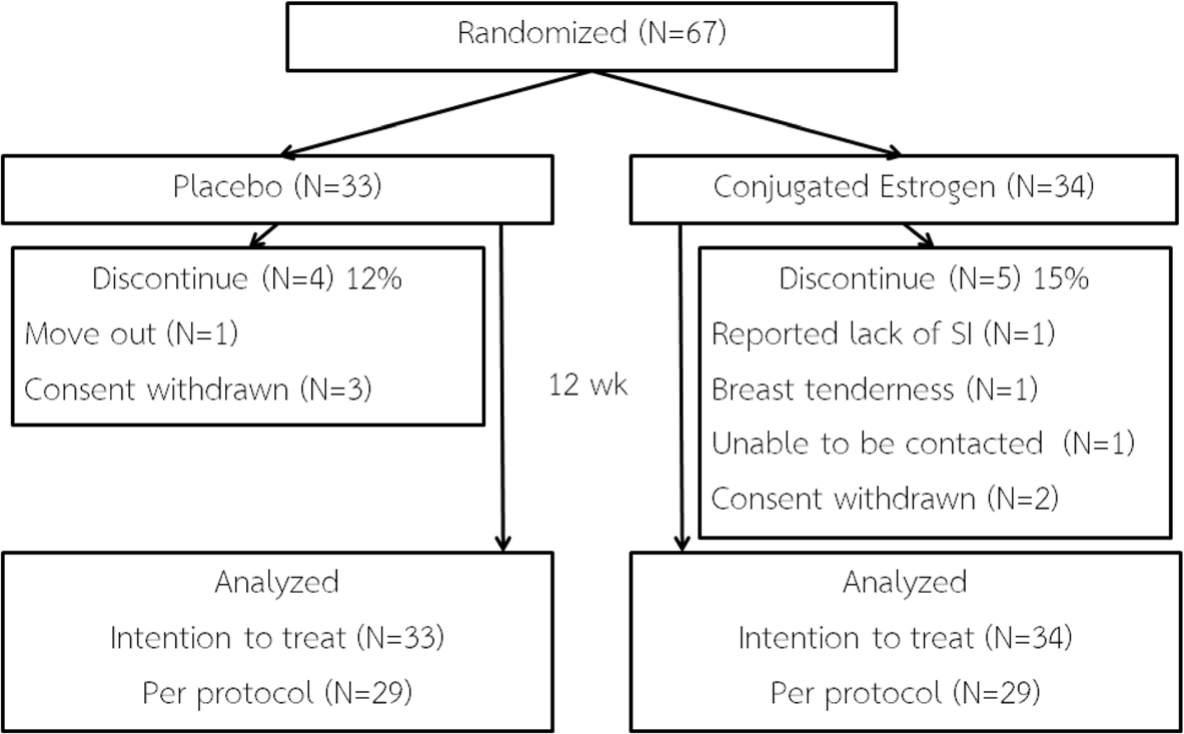
Protocol flow diagram

From August,10th 2018 to March, 18th 2019, a total of 67 participants were enrolled in the study. The enrollment was stopped when the number of participants meet the calculated sample size. Thirty-four participants were randomized into the estrogens arm while another 33 were randomized into the placebo arm (Fig. 1). Fifty-eight participants completed the study, all with compliance of > 90% based on pills count on follow up visit. Frequency of sexual intercourses during 12 weeks was reported to be more than once monthly in both groups. Nine participants, 5 in the estrogens arm and 4 in the placebo arm, discontinued from the study during the 12-week protocol. In those who discontinued participating in the treatment arm, two participants change their mind due to concern about the effect of the treatment, one participant reported breast tenderness and withdrawn consent, one participant reported lack of sexual intercourse which met the exclusion criteria, and one participant lost the follow up visit with no response from available contacts. In those who discontinued participating in the placebo arm, three participants change their mind due to concern about the effect of the treatment and withdrawn consent, and one participant move out of the country and cannot return for the second visit. Demographic data and baseline characteristics were comparable with no statistically differences between the two arms of the study (Table 2); with the mean age of participants at 57.41 ± 4.85 and 57.03 ± 4.65 years; and mean body mass index of 24.62 ± 3.48 and 25.09 ± 3.97 kg/m^2^ in the estrogens and placebo arms, respectively.

**Table 2:** Demographic data and baseline characteristics of all participants (*N* = 67)

***Parameter***	Estrogens group* (n = 34)	***Placebo group*** ***(n = 33)***
Age (years)^a^	57.41 ± 4.85	57.03 ± 4.65
Menopausal age (years)^a^	50.74 ± 3.46	50.30 ± 3.04
Year since menopause (years)^a^	6.68 ± 4.52	6.73 ± 4.03
BMI (kg/m^2^)^a^	24.62 ± 3.48	25.09 ± 3.97
Pregnancies (times)^a^	2.32 ± 1.34	2.30 ± 1.31
Deliveries (times)^a^	1.91 ± 1.16	1.88 ± 0.93
Reported sexual intercourse (times/month)^c^	2 (1–3)	3 (1–8)
Education^b^		
Elementary school or less	11 (33.3%)	11 (32.4%)
Secondary school or diploma	14 (41.18%)	13 (39.39%)
Higher education	9 (26.47%)	9 (27.27%)
Times of marriage ^b^		
Once	30 (88.24%)	24 (72.74%)
Twice	4 (11.76%)	9 (27.27%)
Duration of marriage (years)^c^	30 (25.5–33)	30 (22.5–37.5)
Vaginal pH^c^	6 (5.5–8.0)	6 (5.5–6.0)
Vaginal Maturation Value^c^	15 0–50)	15 (1.25–47.5)

*conjugated estrogens

^a^mean ± SD, ^b^number (%), ^c^median (interquartile range, IQR)

### Primary outcome: sexual function

No significant improvement of the Female Sexual Function Index was observed after using conjugated estrogens for 12 weeks compared to placebo in all six domains and overall index (*p* = 0.182). (Table 3).

**Table 3:** Primary and secondary outcomes (Intention-to-treat analysis, *n* = 67)

***Parameter***	Estrogens group* (n = 34)		***Placebo group (n = 33)***		***P-value***
	***Week 0***	***Week 12***	***Week 0***	***Week 12***	
FSFI: Total ^a^	20.31 ± 4.93	25.14 ± 4.74	22.27 ± 5.03	24.32 ± 4.83	0.182
FSFI: Desire ^a^	2.44 ± 0.82	3.12 ± 0.76	2.73 ± 0.65	2.90 ± 0.64	0.218
FSFI: Arousal ^a^	2.72 ± 1.06	3.46 ± 0.97	3.03 ± 1.28	3.25 ± 0.91	0.133
FSFI: Lubrication ^a^	3.46 ± 1.27	4.54 ± 1.16	3.98 ± 1.04	4.20 ± 1.03	0.057
FSFI: Orgasm ^a^	3.93 ± 1.14	4.26 ± 1.24	4.10 ± 1.12	4.62 ± 1.16	0.365
FSFI: Satisfaction ^a^	4.06 ± 1.02	4.63 ± 0.88	4.52 ± 0.85	4.69 ± 0.99	0.219
FSFI: Pain ^a^	3.71 ± 1.87	5.13 ± 0.95	3.90 ± 1.51	4.66 ± 1.05	0.067
Vaginal pH^c^	6.0 (5.5–8.0)	5.0 (4.0–6.0)	6.0 (5.5–6.0)	7.0 (6.0–8.0)	**< 0.001**
Normal Flora Index^c^	5 (3–6)	6 (4–10)	4 (3–6)	6 (4–6)	0.282
Vaginal Maturation Value^c^	15 (0–53.75)	57.5 (38.75–65)	20 (1.25–51.25)	20 (0.63–46.88)	**< 0.001**
Superficial cell^c^	0 (0–1)	0.5 (1.5–3)	0 (0–2.5)	0 (0–0.88)	**< 0.001**
MBS, total^c^	4 (3–6)	2 (0–3)	5 (3–7)	3 (1–4)	0.182
Vaginal dryness^c^	2 (2–3)	1 (0–2)	2 (2–3)	1 (0–2)	0.858
Vaginal/vulvar irritation/itching^c^	0 (0–2)	0 (0–0)	1 (0–2)	0 (0–1)	0.190
vaginal/vulvar soreness^c^	0 (0–1)	0 (0–0)	0 (0–2)	0 (0–1)	0.204
Dyspareunia^c^	2 (1–3)	0 (0–1)	2 (1–2)	1 (0–1)	0.152

*conjugated estrogens

^a^mean ± SD Data was analyzed by ANCOVA, ^b^number (%), ^c^median (interquartile range, IQR) Data was analyzed by Mann-Whitney U test

Data shown in bold indicates statistical significance (*p* < 0.05)

*FSFI* the Female Sexual Function Index, *MBS* Most Bothersome Symptoms

### Secondary outcome: vaginal pH, VMV, NFI and MBS

Vaginal administration of oral conjugated estrogens tablets could statistically significant improve vaginal pH toward more acidity (*p* = < 0.001) with higher Vaginal Maturation Value (*p* = < 0.001) and superficial cells (*p* = < 0.001). (Table 3) There was no significant difference in NFI between both arms (*p* = 0.282). No significant improvement of MBS, both in total scores (*p* = 0.182) and each symptom, was observed. (Table 3).

### Safety parameters

At the end of the study, there were no significant differences in hematocrit, SGOT, SGPT, alkaline phosphatase, total cholesterol, triglycerides, LDL, HDL, estradiol level and endometrial thickness between the two groups after adjusted for baseline parameter. Only triglycerides were found to be higher in the placebo group after the 12-week treatment (*p* = 0.045) (Table 4).

**Table 4:** Safety outcomes (Intention-to-treat analysis, *n* = 67) ^a^

***Parameter***	Estrogens group* (n = 34)		***Placebo group*** ***(n = 33)***		***P-value***
	***Week 0***	***Week 12***	***Week 0***	***Week 12***	
Hematocrit (%)^**a**^	39.42 ± 3.30	38.68 ± 2.51	39.36 ± 3.23	38.63 ± 3.06	0.655
SGOT (unit/L)^**a**^	28.72 ± 12.05	27.10 ± 15.48	23.79 ± 9.72	24.96 ± 9.62	0.390
SGPT (unit/L)^**a**^	29.86 ± 16.90	30.31 ± 20.95	24.33 ± 14.03	29.33 ± 15.74	0.279
ALP (unit/L)^**a**^	77.07 ± 29.42	76.69 ± 22.15	77.75 ± 20.91	79.38 ± 18.25	0.809
Total cholesterol (mg/dL)^**a**^	215.72 ± 37.62	212.93 ± 38.03	215.04 ± 39.35	218.00 ± 38.20	0.733
Triglycerides (mg/dL)^**a**^	123.69 ± 45.37	114.93 ± 52.79	126.71 ± 64.49	162.33 ± 97.69	**0.045**
HDL (mg/dL)^**a**^	57.21 ± 10.52	58.72 ± 12.75	56.63 ± 11.91	54.29 ± 11.41	0.142
LDL (mg/dL)^**a**^	134.00 ± 34.56	131.17 ± 33.37	133.13 ± 36.19	131.21 ± 39.52	0.825
Endometrial thickness (mm)^**a**^	3.28 ± 2.03	3.38 ± 1.97	3.44 ± 2.30	3.15 ± 1.72	0.677
Estradiol (pg/mL)^**b**^	5.00 (5.00,11.94)	5.00 (5.00,9.61)	5.00 (5.00,12.96)	5.00 (5.00,9.89)	0.218

*conjugated estrogens

^a^mean ± SD Data was analyzed by ANCOVA,

^b^median (interquartile range, IQR) Data was analyzed by Mann-Whitney U test

Data shown in bold indicates statistical significance (*p* < 0.05)

The number of participants reporting adverse events was comparable between the estrogens (51.72%) and placebo arms (41.38%) (Table 5). The most common adverse event in the estrogens arm was breast tenderness (31.03%) of which prompted two participants to withdraw from the study. Surprisingly, the most common adverse event in the placebo arm was also breast tenderness (28.00%). One participant who reported no adverse event on the third week of follow-up phone call could not be contacted at the end of the study.

**Table 5:** Adverse event [number (%)]

***Parameter***	Estrogens group* (n = 29)	***Placebo group*** ***(n = 29)***
Any adverse events	15 (51.72%)	12 (41.38%)
Breast tenderness	9 (31.03%)	7 (28.00%)
Vaginal discharge	4 (13.79%)	4 (13.79%)
Insoluble pill	7 (24.14%)	4 (13.79%)
New onset of vaginal itching	4 (13.79%)	0 (0%)

*conjugated estrogens

## Discussion

This was a 12-week double-blind, randomized, placebo-controlled trial designed to evaluate the effects of vaginal administration of conjugated estrogens tablet on sexual function in postmenopausal women with female sexual disorders (FSD). The results showed that vaginal administration of conjugated estrogens tablet significantly improved vaginal pH and Vaginal Maturation Value, toward more superficial cells. However, there was no significant difference in the Female Sexual Function Index (FSFI) after 12 weeks between the two groups in each domain and overall index. Also there was no significant difference in Normal Flora Index (NFI), and the Most Bothersome Symptoms (MBS) between the two groups.

The treatment drugs, conjugated estrogens tablet (EstromonTM, 0.625 mg), is a combination of *Cynanchum wilfordii* Hemsley, *Phlomis umbrosa* Turczaninow, and *Angelica gigas* Nakai extracts (CPAE). A one-year clinical study in South Korea [31] and another randomized, double-blinded, placebo-controlled clinical study in non-Asian American women [32] confirmed that the formula, administered orally, significantly ameliorated various menopausal symptoms without any serious side effects, increase in body weight, body mass index (BMI) or changes of serum levels of estradiol (E2), follicle stimulating hormone (FSH) and liver enzymes.

Several oral medications have been off-label administered vaginally to treat conditions when the oral route was intolerable. These include misoprostol for induction of labor, cervical ripening, and pregnancy termination; sildenafil to increase blood flow to the uterus in preparation for embryo implantation; bromocriptine for treatment of prolactinoma in those intolerant of nausea/vomiting side effects; oral contraceptives [33–35] and oral hormone therapy preparations for those with intolerable side effects from oral administration [36]. Advantages of the vaginal route include avoidance of the hepatic first-pass effect, thus enabling lower dose administration. The vaginal absorption is unaffected by and can also avoid gastrointestinal disturbances. Less frequent administrations are required than the oral route [37].

Two factors which determine drug absorption from vagina include drug dissolvability and vaginal membrane penetration. These steps are influenced by vaginal physiological factors and physicochemical properties of drugs [38]. Vaginal estrogen was found to be better absorbed by thinner mucosa in postmenopausal women [39]. In addition, the estrogenization of the vaginal mucosa, evidenced from the increase of VMV toward more presence of superficial cells in this study, could also improve absorption of hormones through the vaginal wall [40, 41]. Nevertheless, data was very limited for the relationship between drug physicochemical properties and the human vaginal permeability. As a matter of fact, CPAE with its lipophilic property may have better affinity for vaginal absorption.

There were several studies demonstrated beneficial effects of estrogens, specifically designed to be administered vaginally, on female sexual function and quality of life [42, 43]. This study was designed to be a pioneer of using oral medication, off-labelly administered vaginally to treat FSD. Though the vaginal conjugated estrogens in this study could significantly decrease vaginal pH and improved VMV toward superficial cell domination, there was no statistically significant difference between the two groups in FSFI, NFI and MBS. This is probably due to the fact that sexual function, particularly during postmenopause, is multifactorial. Biological, psychological factors and relationship with partner may have complex interplay on sexual outcome. The use of vaginal estrogens to improve biological conditions of the vagina might not be able to reveal overall effects on clinical indicators such as FSFI and MBS. On the other hand, our pilot study for sample size calculation may not be homogenous with the studied population to render a meaningful result in this study. Moreover, the insignificant improvement of our primary outcome might be due to less-than-expected absorption of the oral estrogen tablet in vagina. Lastly, the switching to longer interval of vaginal estrogens administration after 3 weeks may compromise any discernible clinical outcome which may result from the treatment effect if it does exist. Thus, more often administration of vaginal estrogens or longer duration of the treatment might result in balanced vaginal microbiome, decreased likelihood of GSM, improvement of sexual function and better quality of life in postmenopausal women with female sexual dysfunction (FSD).

For safety concerning potential adverse estrogenic activity of the CPAE, a study [44] in a stably transfected transcriptionally activated human estrogen receptor (hER)-HeLa9903 cell model showed no significant selective activity against hERα and hERβ. The study also showed that CPAE did not increase uterine wet weight in ovariectomized rats, and did not significantly induce MCF-7 cell proliferation, compared with the effects of the positive control E2. The MCF-7 cell line was known to be a hormone-dependent breast cancer cell line that expresses both ERα and ERß. This implied that CPAE was less likely to affect the development of E2-mediated breast cancer cell line.

At the end of the study, there were no statistically significant differences in hematocrit, SGOT, SGPT, alkaline phosphatase, total cholesterol, LDL, HDL, estradiol level and endometrial thickness between the two groups. Only triglycerides were observed to be statistically gained in the placebo group after 12-week treatment. This is not well understood but it might be a coincidence because this is unlikely to be caused by placebo effect.

The reported adverse events were comparable between the two groups. The most common adverse event in the estrogens arm was breast tenderness (33%) which prompted two participants to discontinue the study. This was probably due to the rising of circulating estrogen levels from significant vaginal absorption in some particular individual. Likewise, this may be placebo effect which was also shown in the participants of the placebo arm who also experienced breast tenderness in 25%. Nonetheless, breast tenderness was reported only at the beginning of the study and subsided after 3 weeks when the vaginal administration frequency decreased. Overall, there were no statistically significant changes in systemic estradiol level and endometrial thickness.

The strengths of this study include 1) the nature of the double-blind, randomized, placebo-controlled trial which has advantages in minimizing placebo effect 2) the standardized FSFI questionnaire which is widely used for sexual function studies of which the results could be compared 3) this study recruited participants having both objective (baseline pH > 5) and subjective criteria (symptomatic vaginal atrophy) which were indicated for treatment. 4) Though there were no significant differences in overall clinical indicators such as FSFI and MBS, the differences in certain objective indicators such as VMV and vaginal pH are encouraging evidence that these low-cost and widely available estrogens may be another treatment option for the GSM if there are more confirmative future studies.

However, there are also some limitations in this study which need to be considered 1) since the study period was limited, the safety and efficacy beyond 12 weeks could not be evaluated 2) since the frequency of vaginal administration was switched from daily to twice weekly after 3 weeks, this might obscure the effects of estrogens when compared to placebo at the end of the 12-week study.

Future double-blind, randomized, placebo-controlled trials with sizable sample of postmenopausal women with GSM are needed. This is to minimize the confounding effects of psychological and partnership factors over the effects of GSM on sexual function indicators.

## Conclusion

The 12-week study with vaginal administration of conjugated estrogens tablet had no demonstrable effects on the changes in the Female Sexual Function Index (FSFI), Normal Flora Index (NFI), and Most Bothersome Symptoms (MBS) in postmenopausal women with female sexual dysfunction (FSD). However, the conjugated estrogens usage in aforementioned protocol were found to improve vaginal pH and Vaginal Maturation Value toward superficial cells domination. This 12-week use of conjugated estrogens appeared to have few side-effects. More frequent administration or longer duration of the treatment might be required to improve FSFI and MBS in postmenopausal women with female sexual dysfunction (FSD).

## Data Availability

The datasets used and/or analyzed during the current study are available from the corresponding author on reasonable request.

## Abbreviations

BMI: Body Mass Index
E2: Estradiol
FSD: Female Sexual Dysfunction
FSFI: Female Sexual Function Index
FSH: Follicle Stimulating Hormone
FSIAD: Female Sexual Interest/Arousal Disorders
GPPPD: Genitopelvic Pain/Penetration Disorder
GSM: Genitourinary Syndrome of Menopause
HDL: High-density Lipoprotein
hER: Human Estrogen Receptor
IQR: Interquartile Range
LDL: Low-density Lipoprotein
MBS: Most Bothersome Symptoms
MHT: Menopausal Hormone Therapy
NFI: Normal Flora Index
SD: Standard Deviation
SGOT: Serum Glutamic Oxaloacetic Transaminase
SGPT: Serum Glutamic Pyruvic Transaminase
VMV: Vaginal Maturation Value
VVA: Vulvovaginal Atrophy
